# A robust AFM-based method for locally measuring the elasticity of samples

**DOI:** 10.3762/bjnano.9.1

**Published:** 2018-01-02

**Authors:** Alexandre Bubendorf, Stefan Walheim, Thomas Schimmel, Ernst Meyer

**Affiliations:** 1 Department of Physics, University of Basel, Klingelbergstrasse 82, 4056 Basel, Switzerland; 2 Institute of Nanotechnology (INT) and Institute of Applied Physics (Karlsruhe Institute of Technology (KIT)), Karlsruhe, Germany; 3 Karlsruhe Nano Micro Facility (KNMF), Karlsruhe, Germany

**Keywords:** atomic force microscopy, contact resonances, elastic modulus, 1*H*,1*H*,2*H*,2*H*-perfluorodecyltrichlorosilane (FDTS), polymers, Young’s modulus

## Abstract

Investigation of the local sample elasticity is of high importance in many scientific domains. In 2014, Herruzo et al. published a new method based on frequency-modulation atomic force microscopy to locally determine the elasticity of samples (*Nat. Commun. ***2014**, *5,* 3126). This method gives evidence for the linearity of the relation between the frequency shift of the cantilever first flexural mode Δ*f*_1_ and the square of the frequency shift of the second flexural mode Δ*f*_2_^2^. In the present work, we showed that a similar linear relation exists when measuring in contact mode with a certain load *F_N_* and propose a new method for determining the elastic modulus of samples from this relation. The measurements were performed in non-dry air at ambient temperature on three different polymers (polystyrene, polypropylene and linear low-density polyethylene) and a self-assembled monolayer of 1*H*,1*H*,2*H*,2*H*-perfluorodecyltrichlorosilane (FDTS) on a silicon oxide substrate perforated with circular holes prepared by polymer blend lithography. For all samples the relation was evidenced by recording Δ*f*_1_, Δ*f*_2_ and *F_N_* as a function of the *Z*-displacement curves of the piezoelectric scanner. The occurence of a plastic deformation followed by an elastic deformation is shown and explained. The necessary load *F_N_* for measuring in the elastic domain was assessed for each sample, used for mapping the frequency shifts Δ*f*_1_ and Δ*f*_2_ and for determining the elastic modulus from Δ*f*_2_^2^/Δ*f*_1_. The method was used to give an estimate of the Young’s modulus of the FDTS thin film.

## Introduction

Knowledge of the local elasticity of samples is of high interest in many scientific domains, as many processes and physical quantities are correlated with the elastic modulus. In biology, for instance, studies showed that the elasticity of cells depends on their age, the stage of the cell cycle and the degree of differentiation [1]. In physics, the band gap size of nanocrystals and the presence of planar defects on nanotubes are a function of the Young’s modulus [2–3]. Probing local elasticity requires an instrumentation capable of operating with high resolution and under different conditions, such as variable temperature, pressure or humidity. Since its invention, the atomic force microscope (AFM) [4] has confirmed its value for locally determining nanomechanical properties, such as the Young’s modulus, of the sample surface. Initially, the measures were done qualitatively, with the cantilever operated in intermittent-contact mode by showing the phase-shift contrast between regions of different elasticities [5]. This was followed by quantitative measurements using various static and dynamic methods [6–7]. Although the results obtained with these methods are in good agreement with theoretical data and data obtained from macroscopical experiments, difficulties in the precise determination of the elastic modulus based on the theoretical model, or during the use of the method may be encountered when using dynamic-mode AFM. This is the case with the methods devised by Hurley and Turner [6] and Herruzo and co-workers [7]. In Hurley and Turner’s [6] method, the stated equations for the computation of the normal sample stiffness by numerical methods (analytical expression for normal sample stiffness formulated by Bubendorf [8] and given in Supporting Information File 1) used to determine sample elasticity are based on the equations established by Rabe [9] and Rabe et al. [10] for atomic force acoustic microscopy (AFAM) [11–14]. They describe the dynamics of a clamped cantilever elastically coupled with the sample surface at its tip end. These equations have the disadvantage of strongly depending on the dimensions of an ideal beam-shaped cantilever. However, most cantilevers used for measurements are not ideal. Thus, to achieve consistent results, the values of length and tip height have to be corrected. When measurements using the multifrequency AFM [15–16] method of Herruzo et al., which is based on the excitation of two cantilever eigenmodes [17–21], are performed in non-dry air, the instability of the tip–sample distance feedback loop, due to the use of the frequency shift as control parameter, makes the application of the method difficult if not impossible. However, despite these disadvantages, both methods are particularly interesting because of the complementarity of their advantages. The method of Hurley and Turner [6], which is based on tracking the first flexural and torsional contact resonances, has the advantage of staying stable even if the measurements are performed in non-dry air. In contrast, the method of Herruzo et al. [7] uses a simple theoretical model that depends only weakly on the dimensions of the cantilever. We present a new dynamic method for measuring the sample elasticity [8] that combines the simplicity of the theoretical model of Herruzo et al. [7] with the robustness of the measuring method based on contact resonances. Since the range of elasticity values of polymers is covered by the domain of validity of the theoretical model, those values were used for testing the new method.

## Principle of the Method

### Theoretical model

The method is based on the theoretical model developed by Herruzo et al. [7] for the computation of the effective elastic modulus of samples *E*_eff_ ranging from 1 MPa to 3 GPa from the measured frequency shifts of the two flexural modes of a cantilever operated in intermittent-contact mode:

[1]
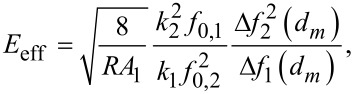


where *R* is the effective tip radius and *k_i_*, *A_i_*, *f_0,i_* and Δ*f_i_*(*d_m_*) are, respectively, the force constant, the oscillation amplitude, the resonance frequency and the frequency shift of the *i*-th flexural mode in free space as a function of *d_m_*, the closest distance between tip and sample in an oscillation cycle.

The validity of the relation and the stability of the microscope during its operation is ensured by the use of an amplitude *A*_2_ small enough compared to *A*_1_ (ideally, *A*_1_ is at least one order of magnitude larger than *A*_2_). The applied normal force on the sample is controlled by controlling Δ*f*_1_, so that Δ*f*_2_ changes as a function of sample elasticity. In our method based on tracking the contact resonances, the applied normal force *F_N_* is directly controlled by controlling the vertical deflection of the cantilever. We can hence rewrite the previous equation as

[2]
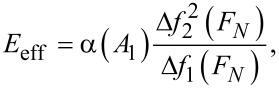


where α is a parameter that depends on amplitude *A*_1_, determined from a sample of known elastic modulus. Δ*f*_1_ and Δ*f*_2_ are the frequency shifts that depend on the applied normal force, *F_N_*. They are determined from the measured contact resonances through the relation Δ*f_i_* = *f_i_* − *f_0,i_*, where *f_i_* is the contact resonance of the *i*-th flexural mode.

Herruzo et al. [7] showed that if the Young’s modulus of the tip *E*_tip_ is at least two orders of magnitude larger than that of the sample *E*_sample_ then

[3]
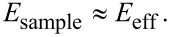


### Feedback controls

**Figure 1:**
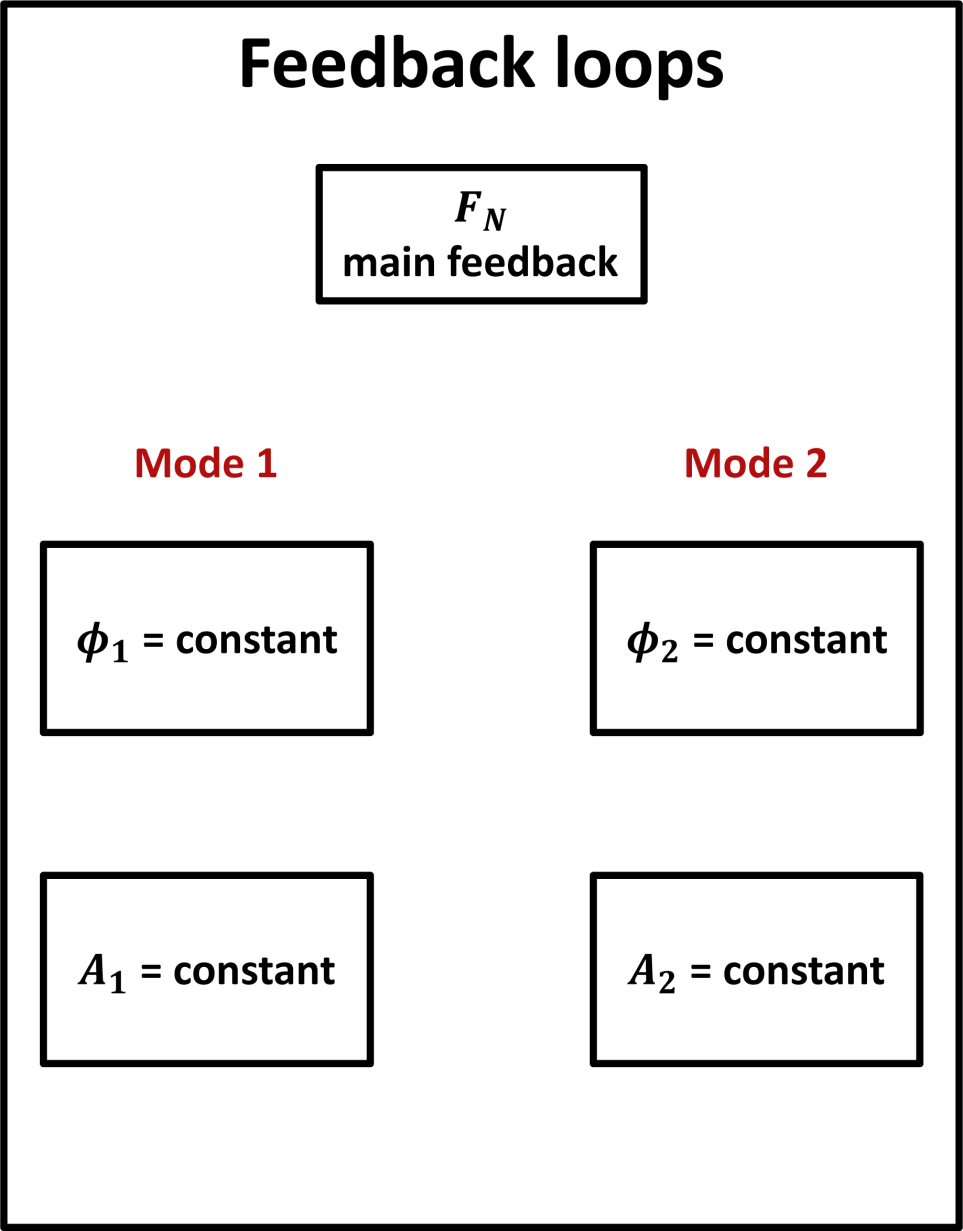
Feedback loops necessary to track the flexural contact resonances of the cantilever. The main feedback loop ensures that the measurements are performed with a constant normal force. The other feedback loops are used to maintain constant values of phase 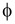
_1_ and amplitude *A*_1_ of the first flexural mode, and of phase 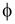
_2_ and amplitude *A*_2_ of the second flexural mode.

As introduced by Herruzo et al. [7], five different feedback loops are used as feedback controls, as shown in Figure 1: two feedback loops for keeping the amplitudes *A*_1_ and *A*_2_ constant, two feedback loops for keeping the phase shifts 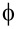
_1_ and 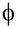
_2_ constant in order to track the contact resonances *f*_1_ and *f*_2_, and the last feedback loop as main feedback for controlling the applied normal force *F_N_*.

## Experimental

### Microscope and data acquisition

The measurements were performed with a flex AFM head from Nanosurf and the Nanonis scanning probe microscope controller. The system integrates the phase-locked loops (PLLs) necessary for tracking the contact resonances and all the controlling and signal generation modules for measuring and mapping the physical quantities. Square areas of 2.5 μm × 2.5 μm were scanned at a resolution of 256 × 256 pixels and a scan speed of 2 μm·s^−1^.

### Cantilevers

The measurements were performed with a PPP-CONT cantilever from Nanosensors^TM^ (Si tip of Young’s modulus *E*_tip_ ≈ 169 GPa) characterized by *f_0,1_* = 16.35 kHz, *k*_1_ = 0.324 N·m^−1^, *Q*_1_ = 68, *f_0,2_* = 102.98 kHz, *k*_2_ = 13.1 N·m^−1^, *Q*_2_ = 208, where *Q_i_* is the *Q* factor of the *i*-th flexural mode in free space. The force constants were computed for *k*_1_ from the theoretical formula *k*_1_ = (*Ewt*^3^)/(4*l*^3^) [22], where *E*, *t*, *w* and *l* are, respectively, Young’s modulus, thickness, width and length of the cantilever beam, and for *k*_2_ by using the relation *k*_2_ = 40.4*k*_1_ established by Rast and co-workers [23]. The thickness *t* = 2.35 μm was determined from *f_0,1_* and the formula *t* ≈ 6.1911 (ρ/*E*)^(1/2)^*f_0,1_l*^2^ [22]. Width *w* = 52 μm and length *l* = 445 μm of the cantilever were determined by optical microscopy. The values *E =* 169 GPa and ρ = 2,330 kg·m^−3^ were taken for the Young’s modulus of the silicon beam and its mass density. The measured normal force *F_N_*_,meas_, which corresponds to the vertical deflection of the cantilever and gives the value of the applied normal force *F_N_* on the sample surface was calibrated with a factor obtained by dividing the cantilever spring constant by the cantilever optical sensitivity *S_z_*. The optical sensitivity of value 229 nm·*V*^−1^ was determined by measuring a vertical deflection–distance curve on an n-type silicon(111) sample of electrical resistivity = 10 Ω·m and by taking the inverse of the slope. The two first flexural modes of the cantilever in contact were excited with the amplitudes *A*_1_ = 22 mV and *A*_2_ = 5 mV for all samples.

### Samples and measurement conditions

Three different polymers (polystyrene (PS), polypropylene (PP) and linear low-density polyethylene (LLDPE)) and a self-assembled monolayer (SAM) of 1*H*,1*H*,2*H*,2*H*-perfluorodecyltrichlorosilane (FDTS) on a silicon oxide (SiO*_x_*) substrate were investigated. The SAM was prepared with circular holes obtained by polymer blend lithography (PBL) [24]. A reference sample consisting of polytetrafluoroethylene (PTFE), commonly called Teflon, with a nominal Young’s modulus of 500 MPa was used to determine parameter α(*A*_1_). The measurements were performed in non-dry air with a relative humidity of 36% at an ambient temperature of 27 °C.

## Results and Discussion

### Force–distance and frequency shift–distance curves

**Figure 2:**
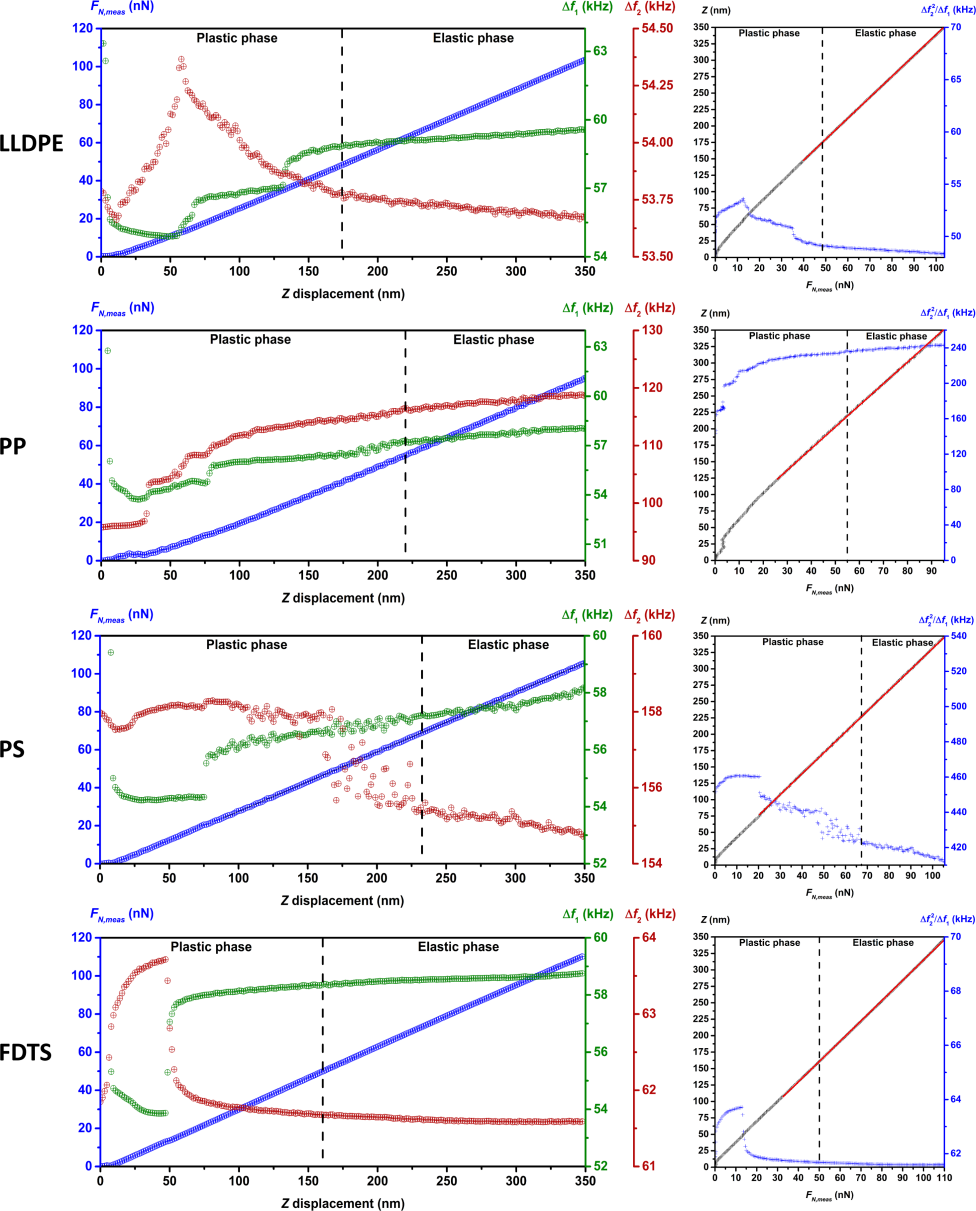
Left: Force–distance and frequency shift–distance curves of LLDPE, PP, PS and FDTS for the determination of the minimal normal force *F_N_* used as setpoint for the elasticity measurements. The curves for Δ*f*_1_ and Δ*f*_2_ as functions of the *Z*-displacement evidence the existence of a plastic deformation phase followed by an elastic deformation phase. Right: The fitting of the *Z*(*F_N_*_,meas_) curves gave a value close to the cantilever spring constant showing the excessive compliance of the cantilever. The 
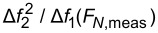
 curves show quasi-invariance of the ratio 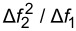 in the elastic deformation phase and in the highest region of the plastic deformation phase.

**Figure 3:**
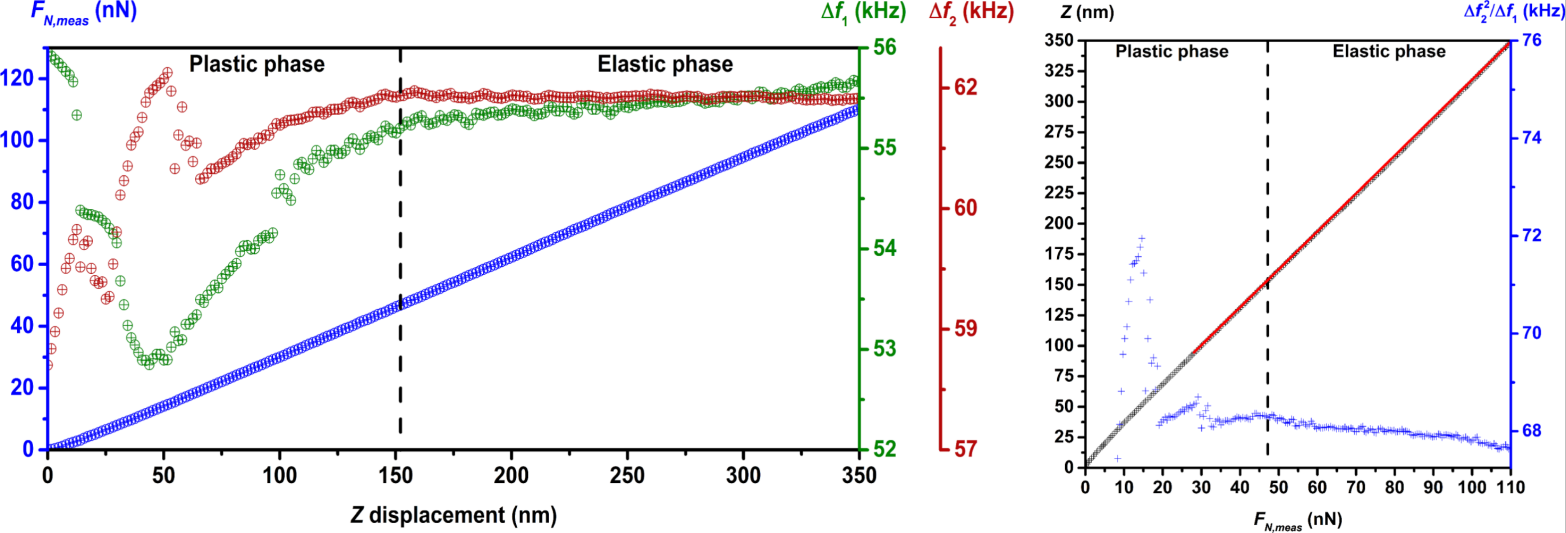
Left: Force–distance and frequency shift–distance curves of PTFE. The curves of Δ*f*_1_ and Δ*f*_2_ as a function of the *Z*-displacement evidence the existence of a plastic deformation phase followed by an elastic deformation phase. Right: The fitting of the *Z*(*F_N_*_,meas_) curve gave a value close to the cantilever spring constant showing the excessive compliance of the cantilever. The 
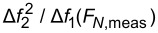
 curves show quasi-invariance of the ratio 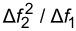 in the elastic deformation phase and in the highest region of the plastic deformation phase. The parameter α(*A*_1_) determined by dividing the nominal value of the elasticity of the reference sample by the mean value of the ratio in the elastic deformation phase gave a value of 7,355 Pa·Hz^−1^.

Before starting the investigation of the sample elasticity, a force–distance curve and a frequency shift–distance curve (Figure 2 and Figure 3, left) for both flexural modes were recorded for each sample to determine for which values of *F_N_* the relation between Δ*f*_1_ and 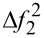 is linear and to compute the parameter α(*A*_1_). The measured applied normal force *F_N_*_,meas_ and frequency shifts Δ*f*_1_, Δ*f*_2_ were recorded for a displacement of the piezoelectric scanner in the normal *Z*-direction from 0 to 350 nm. To measure the curves, the cantilever tip first indented the sample to a depth corresponding to a displacement of the *Z*-scanner of 350 nm and both PLLs were then switched on. The curves were recorded during retraction of the tip in order to avoid unlocking the PLLs. This would happen if the tip indented the sample from a starting position out of contact, as contact resonances are quite far from those in free space.

In the frequency shift–displacement curves, we observe first a nonlinear relation between the measured frequency shifts, Δ*f*_1_ and Δ*f*_2_, and the displacement of the scanner, followed by a linear relation. This suggests a plastic deformation phase of the sample surface during the first step of the indentation, followed by an elastic deformation phase. Analysis of the force–displacement curve evidences the same relations with, however, a linear relation in the nonlinear domain of the frequency shift–displacement curves. This is explained by the low spring constant of the cantilever in comparison to the normal sample stiffness, beginning at a certain *Z*-displacement value.

**Figure 4:**
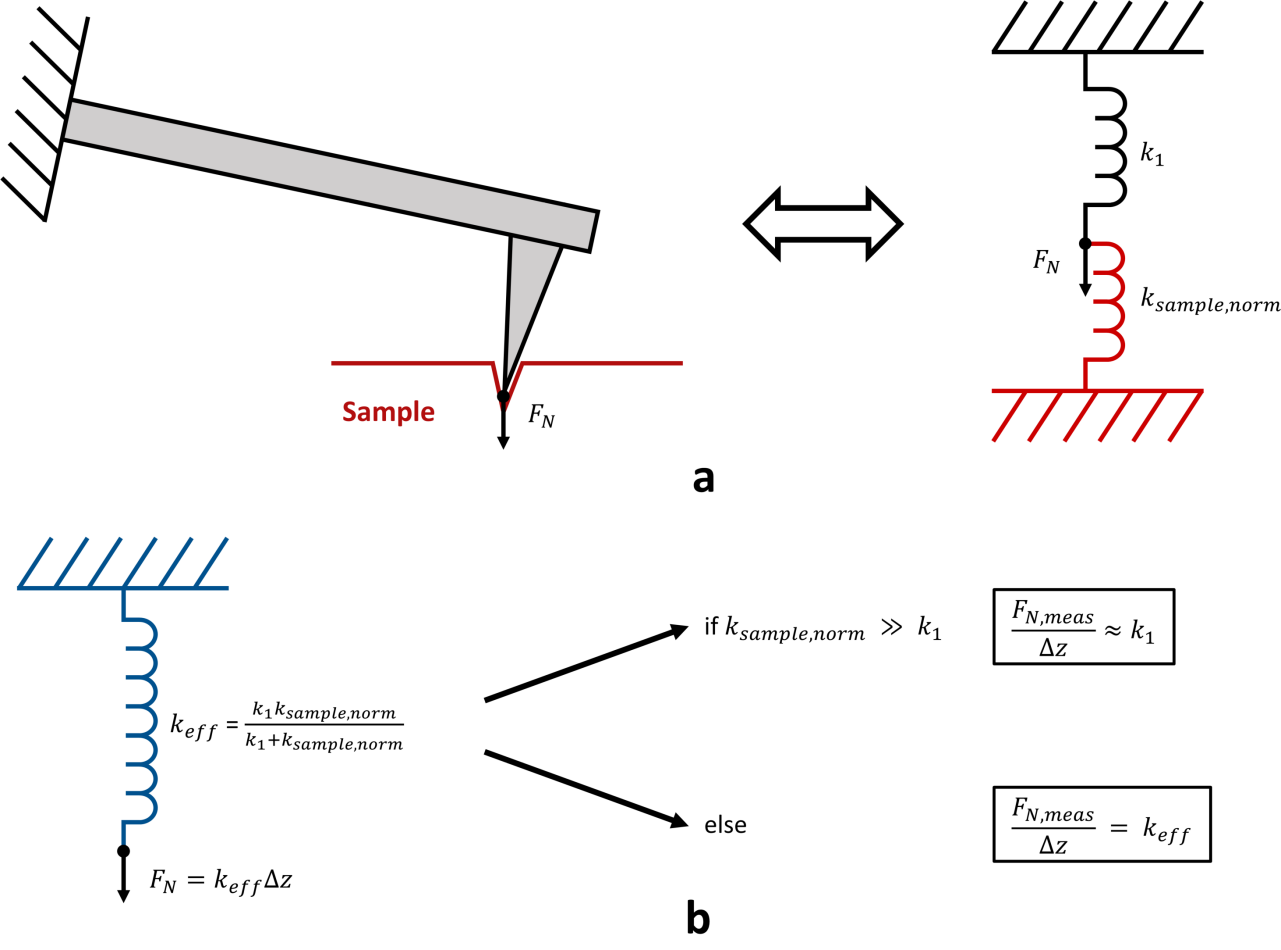
(a) Cantilever in contact with the sample surface modeled by two springs in series, *k*_1_ and *k*_sample,norm_, representing the cantilever spring constant and the normal sample stiffness, respectively. The arrow *F_N_* represents the applied normal force. (b) Effective spring constant *k*_eff_ and values of the ratio between measured applied normal force *F_N_*_,meas_ and *Z*-displacement Δ*z* as a function of the sample stiffness.

A good model of the cantilever in contact with the sample surface is two springs in series, *k*_1_ and *k*_sample,norm_, representing the spring constant of the cantilever and the normal sample stiffness (of constant value in the elastic phase), respectively (Figure 4a). As shown in Figure 4b, the applied normal force generated by the *Z*-displacement Δ*z*, is *F_N_* = *k*_eff_Δ*z*, where *k*_eff_ is the effective spring of value *k*_1_*k*_sample,norm_/(*k*_1_ + *k*_sample,norm_). As the normal stiffness of sample increases during indentation, the value of *k*_eff_ approaches *k*_1_, so that beyond a certain value of *k*_sample,norm_, the variation of *k*_sample,norm_ cannot be observed anymore in the *F_N_*,meas(*Z*) curve. This effect is evidenced in Figure 2 and Figure 3 (right) with the *F_N_*,meas(*Z*) curve (black dots), where the inverse of the slope of the linear fit (red line) shows a value close to the cantilever spring constant as given in Table 1.

**Table 1:** Value of the inverse of the fitting curve slope of *Z*(*F_N_*_,meas_) for the measured samples in Figure 2 and Figure 3 (right). The values are close to the cantilever spring constant.

sample	inverse of slope (N·m^−1^)

LLDPE	0.315
PP	0.308
PS	0.313
FDTS	0.324
PTFE	0.320

### Sample surface deformations and evolution of stress during indentation

**Figure 5:**
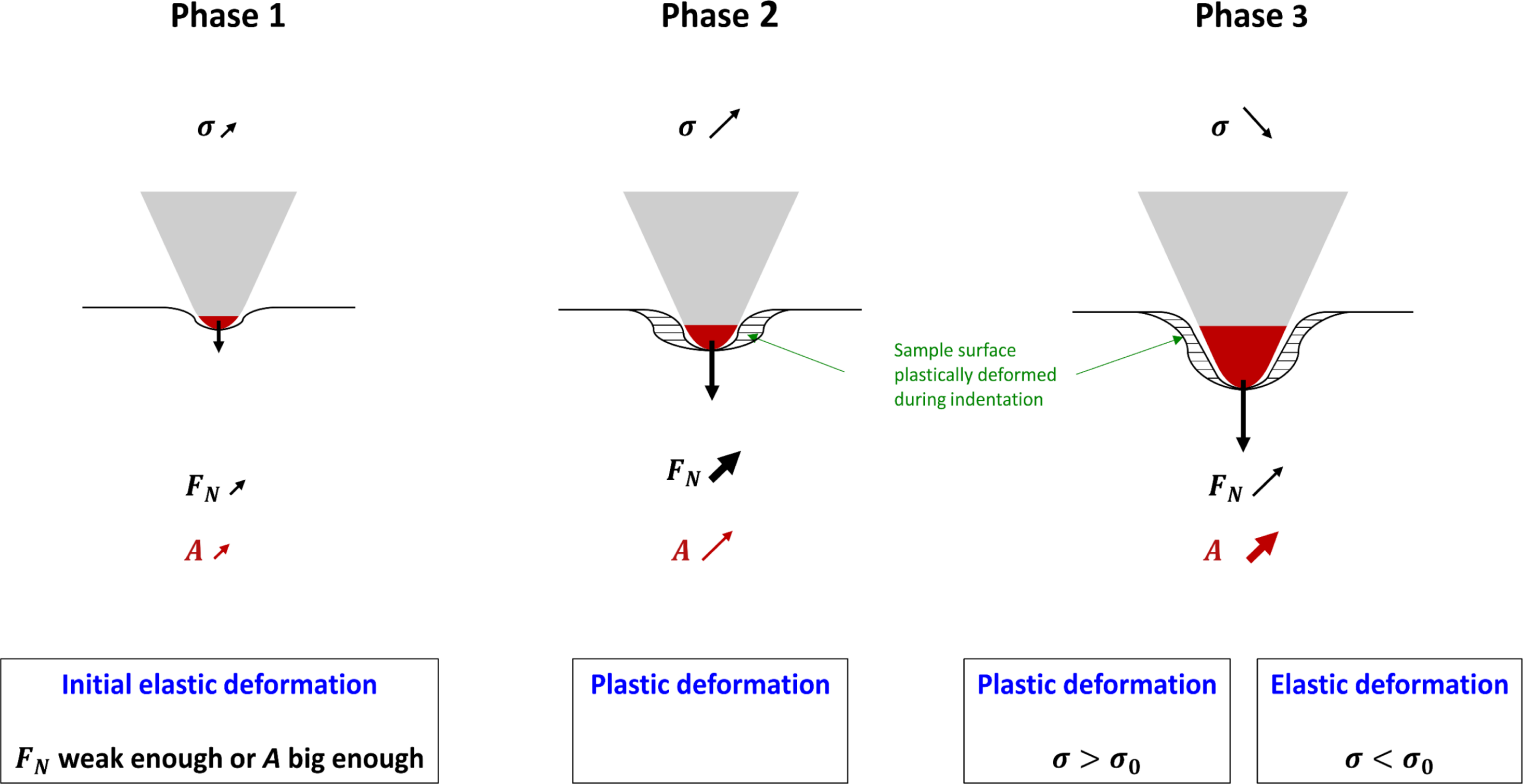
Three phases characterizing the evolution of stress σ during indentation. This evolution is responsible for the plastic and elastic deformation of the sample. In phase 1, in the presence of a sufficiently weak applied normal force *F_N_* or a sufficiently large cross section *A*, the generated stress is small enough to deform the surface elastically. This initial elastic phase can only be observed, if the steps used for the *Z*-scanner are small enough. If the steps are too big, the evolution of σ is directly initiated with phase 2. In phase 2, the large value of *F_N_* and its fast increase compared to *A* generate a stress large enough to deform the surface plastically. In phase 3, the geometry of the tip results in a fast increase of the contact area, and hence of *A*, compared to the moderate increase in *F_N_*, which decreases the stress. When the stress decreases below a threshold value σ_0_, the deformation becomes elastic. The red area of the tip apex describes the contact area of the tip. The striped area shows the region of the sample plastically deformed during indentation. The arrow at the tip apex represents the applied normal force *F_N_*; the arrows on the right of σ, *F_N_* and *A* characterize the increase speed (bold for fast) and magnitude (length).

The existence of a plastic and an elastic deformation phase is explained by the evolution of stress σ (ratio between the applied normal force *F_N_* and the cross section *A* of the cantilever tip) during indentation (Figure 5). At the beginning of the indentation, after setting the tip on the sample surface, stress is increased from a normal force with an initial value 0 nN. The evolution of stress can be split into three phases. In the first phase, if the applied normal force increases gently or if the contact surface between the tip apex and the surface is large enough, the generated stress increases gently enough to deform the sample surface elastically. This elastic phase does not occur if the steps of the *Z*-scanner, which are responsible for increasing the applied normal force, are too big. In this case, the generated stress is strong enough to deform the surface plastically, and phase 2 is initiated. In the second phase, due to the assumed pyramidal geometry of the tip, the contact area is small enough and the applied normal force is strong enough to create a stress that deforms the sample surface plastically. In the third phase, the tip geometry and the increase of the indentation depth result in a larger increase in the contact area than that produced by the applied normal force, thus decreasing the stress on the sample surface. When the stress decreases below a threshold value, σ_0_, the sample starts deforming elastically.

### Linear relation between Δ*f*_1_ and Δ*f*_2_^2^ and determination of α(*A*_1_)

The linearity relation between Δ*f*_1_ and 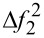 is shown in Figure 2 and Figure 3 (curves on the right) where the quasi-invariance of the ratio 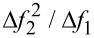 in the elastic deformation phase and in the upper part of the plastic deformation phase can be observed. The mean value of the linear coefficient for each sample and the corresponding maximum deviation in percent are reported in Table 2. The weak deviation in the upper part of the plastic deformation phase suggests that setpoints for *F_N_* in that region can be used to investigate the sample elasticity. The parameter α(*A*_1_) was determined by dividing the nominal value of the Young’s modulus of PTFE by the mean value of 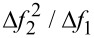 of PTFE. The computation yielded a value of 7,355 Pa·Hz^−1^.

**Table 2:** Mean value of 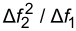 and corresponding maximum deviation (in percent) in the elastic deformation phase and the upper region of the plastic deformation phase.

sample	mean Δ*f*_2_^2^/Δ*f*_1_ (Hz)	max. dev. elastic phase (%)	max. dev. plastic phase (%)

LLDPE	48,720	0.89	3
PP	239,738	1.77	5
PS	417,224	1.38	6.7
FDTS	61,614	0.1	0.8
PTFE	67,981	0.52	1

### Investigation of sample elastic modulus

**Figure 6:**
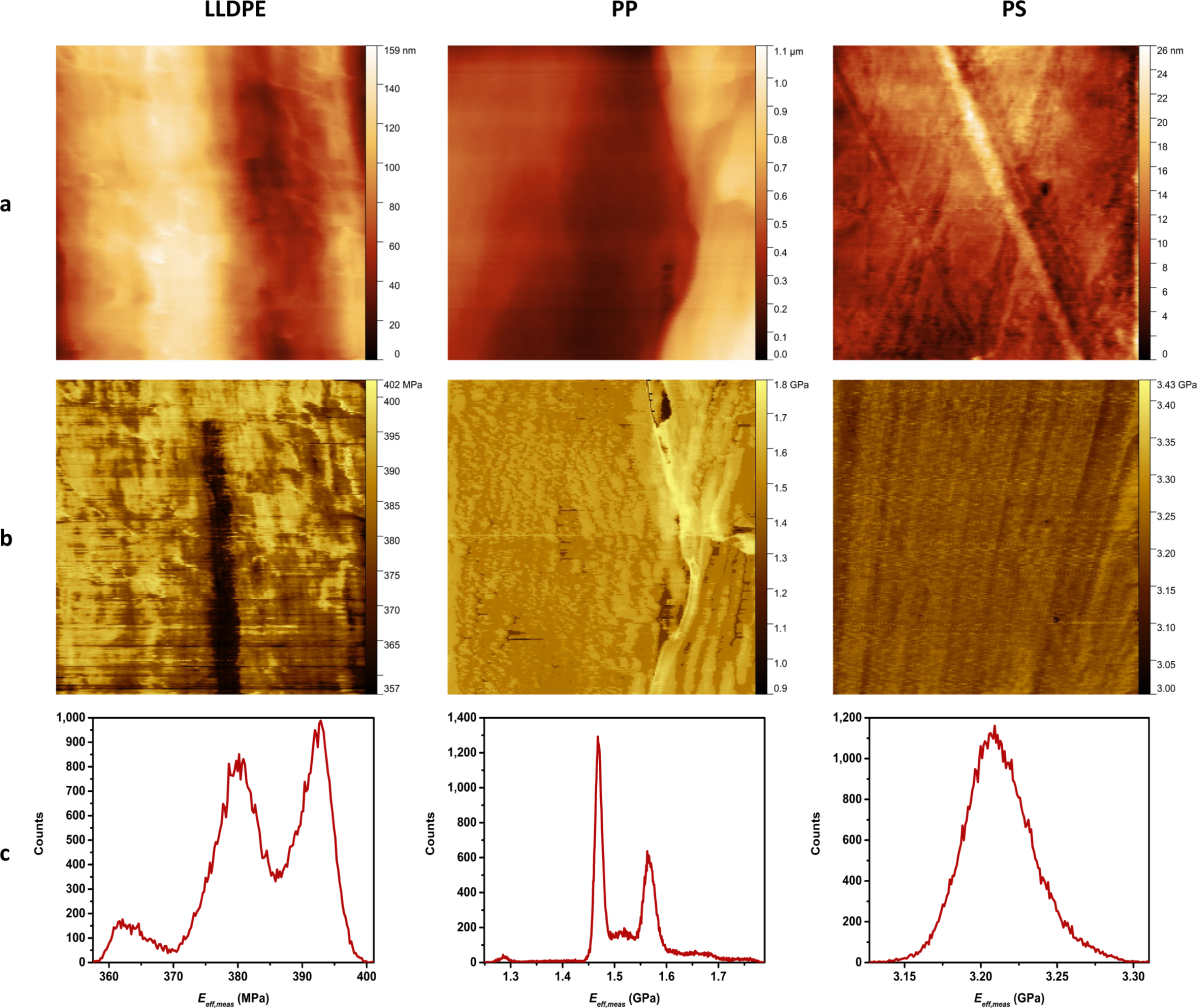
Mapping of (a) topography and (b) elastic modulus of 2.5 μm × 2.5 μm areas of the LLDPE, PP and PS samples. The measurements were performed in contact by applying a constant normal force *F_N_* of 44.4 nN for LLDPE and 29.6 nN for PP and PS, and by exciting the two first flexural modes of the cantilever with an amplitude of *A*_1_ = 22 mV and *A*_2_ = 5 mV, respectively, for the first and second mode. The histograms in panel (c) show the distribution of elastic modulus *E*_eff,meas_ in the maps in panel (b).

**Figure 7:**
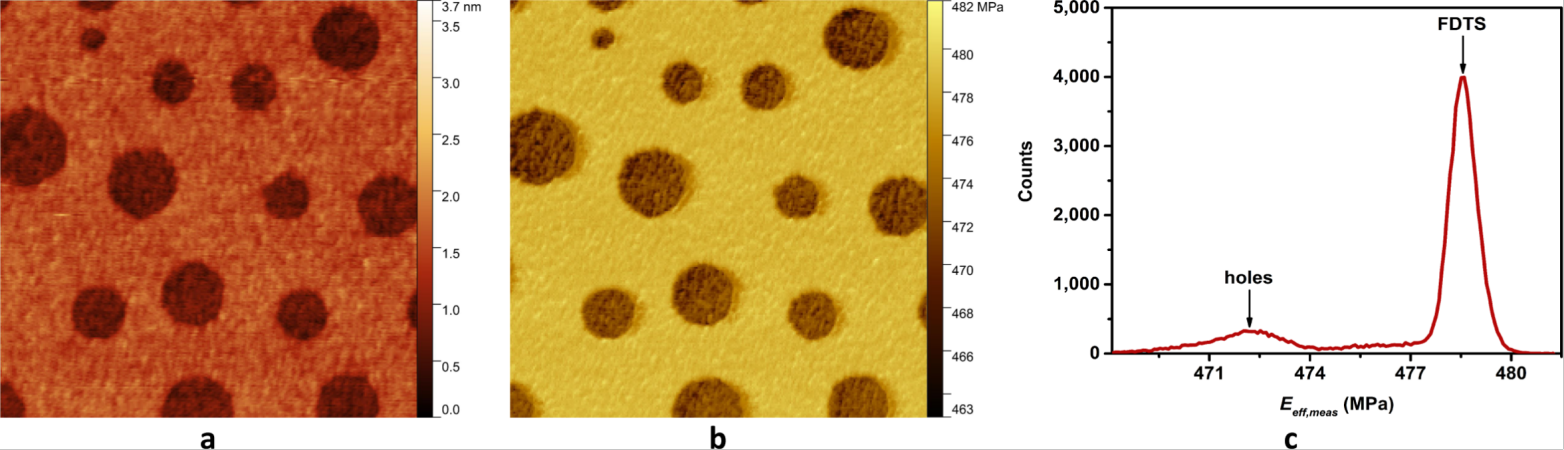
Mapping of (a) topography and (b) elastic modulus of a 2.5 μm × 2.5 μm area of the FDTS + SiOx SAM sample. The measurements were performed in contact by applying a constant normal force *F_N_* of 37 nN, and by exciting the two first flexural modes of the cantilever with an amplitude of *A*_1_ = 22 mV and *A*_2_ = 5 mV respectively for the first and second mode. The histogram in panel (c) shows the distribution of elastic modulus *E*_eff,meas_ in panel (b).

The elastic modulus of the sample was then investigated with setpoints of the main feedback in the highest region of the plastic phase of 44.4 nN for LLDPE, 29.6 nN for PP, 29.6 nN for PS and 37 nN for FDTS. The results of the scannings are shown in Figure 6 and Figure 7 for topography (panel a) and elastic modulus (panel b). The histograms in Figure 6c and Figure 7c correspond to the distribution of the elastic modulus for each map in Figure 6b and Figure 7b. Because polymers are viscoelastic materials, the elastic modulus *E*_eff,meas_ measured on the investigated samples in dynamic mode corresponds to the storage modulus. As the measured contact resonances are quite large compared to the inverse of typical material relaxation time, we can assume that the measured storage modulus is independent on the frequency. The measurements yielded values in the range of the Young’s moduli of bulk LLDPE, PP and PS as seen in Table 3. The investigation also evidenced regions of different elastic moduli, as seen in the histograms of LLDPE, PP and the SAM: LLDPE shows three different peaks centered at 362, 380 and 393 MPa, PP shows two peaks centered at 1.468 GPa and 1.565 GPa and the SAM shows two peaks centered at 472 and 478.5 MPa. Finally, the value of the storage modulus of FDTS gives an estimate of the Young’s modulus of the FDTS monolayer.

**Table 3:** Measured storage moduli *E*_eff,meas_ and literature Young’s modulus values of bulk materials *E*_sample,lit_ of LLDPE, PP, PS and FDTS. The values of *E*_eff,meas_ are in the range of the Young’s moduli of the bulk materials. No literature value is available for FDTS.

sample	*E*_eff,meas_ (GPa)	*E*_sample,lit_ (GPa)

LLDPE	0.362 ± 0.004, 0.38 ± 0.004, 0.393 ± 0.005	0.3–0.7 [25]
PP	1.468 ± 0.009, 1.565 ± 0.015	0.896–1.55 [26]
PS	3.21 ± 0.02	2.28–3.34 [26]
FDTS	0.4785 ± 0.0005	

### Effect of tip functionalization on the SiOx elasticity peak of the SAM sample

**Figure 8:**
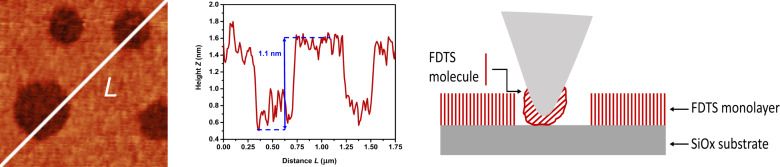
Line profile of the SiOx holes in the topographical mapping of the SAM showing their relative height and width due to the functionalization of the cantilever tip with FDTS material. On the right, a schematic of the tip–sample interaction is depicted. Loosely bound FDTS molecules are able to migrate to the AFM tip and can therefore functionalize by forming a thin layer on the tip apex.

The proximity of the peaks of FDTS and SiOx holes can be explained by the fact that during scanning, some material of FDTS has gathered around the tip, so that the relatively soft FDTS-coating of the tip is sensed (see also Figure 8). The effect of collecting Teflon-like molecules with AFM tips has been known for a long time. It has been successfully used for the topographic imaging of the Si(111) surface with atomic resolution. Howald et al. [27] studied the Si(111) 7 × 7 reconstruction in UHV by contact force microscopy. They observed adhesive forces of up to 103 nN between the Si tip and the Si(111) surface. By coating the tip with PTFE (Teflon), they could reduce the sticking forces to 10 nN. A short scan on a PTFE sample led to a reliable coating of the tip for the subsequent AFM scan on silicon. Since our sample consists of a perforated Teflon-like monolayer, the cantilever tip is most likely constantly functionalized with a thin layer of FDTS. This leads to the observed soft elasticity in- and outside the holes. The stability of this functionalization during the scan can be verified with topographic images. The holes show a depth slightly below 1.2 nm, which is the height of the FDTS monolayer, as seen in Figure 8. If the tip coating was worn-off during the scan, larger or at least partially deeper holes would appear.

## Conclusion

We present a robust method for the quantitative determination of local elastic modulus of sample under ambient conditions (humidity and temperature). It combines the simplicity of the theoretical model developed by Herruzo et al. [7] for the determination of sample elasticity from 1 MPa to 3 GPa with a robust measuring method based on contact resonances. The measurements consist of tracking the first and second flexural contact resonances of the cantilever to determine the frequency shifts Δ*f*_1_ and Δ*f*_2_ relative to the corresponding resonances in free space in order to compute the elastic modulus.

The linear relation between Δ*f*_1_ and 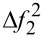 established in [7] can be determined from the measurement of Δ*f*_1_ and Δ*f*_2_ as functions of the *Z*-displacement of the piezoelectric scanner. These curves were measured on four polymers, i.e., LLDPE, PP, PS, PTFE, and a Teflon-like fluorinated SAM.

Analysis of these curves evidenced the existence of plastic and elastic deformation phases. The two subsequent phases are explained by the evolution of stress σ during indentation by the cantilever tip. Plastic deformation begins when sufficient stress is applied to the surface. The slow increase in tip cross section *A* compared to the applied normal force *F_N_* results in an increase in σ and leads to the plastic deformation of the sample surface. The geometrical shape of the tip, assumed to be pyramidal, results in a fast increase in tip cross section compared to the increase in applied normal force *F_N_* when the tip goes deeper into the surface. This, in turn, decreases the stress. When the σ is below a threshold value, the surface begins to deform elastically.

The elastic phase is characterized by a linear relation between the frequency shifts Δ*f*_1_ and Δ*f*_2_ and the *Z*-displacement of the piezoelectric scanner. A linear relation between the measured normal force *F_N_*_,meas_ and the displacement in both phases can also be observed if the spring constant of the cantilever is small compared to the normal sample stiffness. This linearity does not reflect the variation in normal sample stiffness but the excessive compliance of the cantilever. The use of cantilevers with higher spring constants is actually limited for technical reasons. Indeed, for cantilevers with a too high spring constant, the second flexural contact resonance is out of the bandpass of the vertical deflection channel in most of the microscope heads.

The analysis of the 
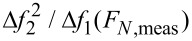
 curve showed that setpoints for the applied normal force in the highest region of the plastic deformation phase could be used without causing a large deviation in the elasticity compared with the results we would obtain in the elastic deformation phase. The elasticity of LLDPE, PP, PS, and the SAM was then investigated. As polymers are viscoelastic materials, the elastic modulus *E*_eff,meas_ measured in dynamic mode corresponds to the storage modulus. We assumed a frequency independence of the measured storage modulus as the measured contact resonances are quite large compared to the inverse of typical material relaxation times. The measurements showed values for the storage modulus in the range of the Young’s moduli for bulk materials for LLDPE, PP and PS.

The method can also distinguish variations in the elasticity of surface such as LLDPE, PP and the FDTS SAM. The investigated SAM consists of a monolayer of FDTS patterned with circular 1.2 nm deep holes. Due to the functionalization of the cantilever tip by SAM molecules, a similar stiffness is measured inside and outside the holes. Finally, the measured value of the storage modulus of 478.5 ± 0.5 MPa, which is close to the value for bulk PTFE (500 MPa), can be used as an estimation of the Young’s modulus of the FDTS monolayer.

## Supporting Information

File 1 Supporting information features the analytical expression for normal sample stiffness based on Hurley and Turner’s equations.
