# Supplementary material for: A robust AFM-based method for locally measuring the elasticity of samples
Source: Beilstein J Nanotechnol. 2018 Jan 2;9:1–10. doi: 10.3762/bjnano.9.1 (PMC5769082; doi:10.3762/bjnano.9.1)
Supplement: File 1 — Supporting information features the analytical expression for normal sample stiffness based on Hurley and Turner’s equations. [file Beilstein_J_Nanotechnol-09-01-s001.pdf]

Supporting Information

for

A robust AFM-based method for locally measuring the elasticity of samples

Alexandre Bubendorf*[1], Stefan Walheim[2,3], Thomas Schimmel[2] and Ernst Meyer[1]

Address: [1]Department of Physics, University of Basel, Klingelbergstrasse 82, 4056 Basel, Switzerland and [2]Institute of Nanotechnology (INT) and Institute of Applied Physics (Karlsruhe Institute of Technology (KIT)), Karlsruhe, Germany and [3]Karlsruhe Nano Micro Facility (KNMF), Karlsruhe, Germany

Email: alexander.bubendorf@outlook.fr

* Corresponding author

Analytical expression for normal sample stiffness

The following computation steps establish the analytical expression for normal sample stiffness [1]. The formula is derived from the equations stated by Hurley and Turner [2] for the numerical determination of normal sample stiffness. These equations, i.e., equations (1), (2), (3), (4), (5), (6) and (7), are based on the description of the dynamics of a clamped, ideally beam-shaped cantilever elastically coupled to a sample by its tip, published by Rabe [3] and Rabe and co-workers [4]. The model takes into account characteristics of the cantilever, such as tilt angle α and dimensions, namely total length L, length from the clamped end to the tip L_1, length from the tip to the free end L_2, tip height h, but also characteristics of the sample, i.e., normal sample stiffness $k_{sample,norm}$ and lateral sample stiffness $k_{sample,lat}$ represented by two springs coupled to the cantilever tip, as shown in Figure S1.

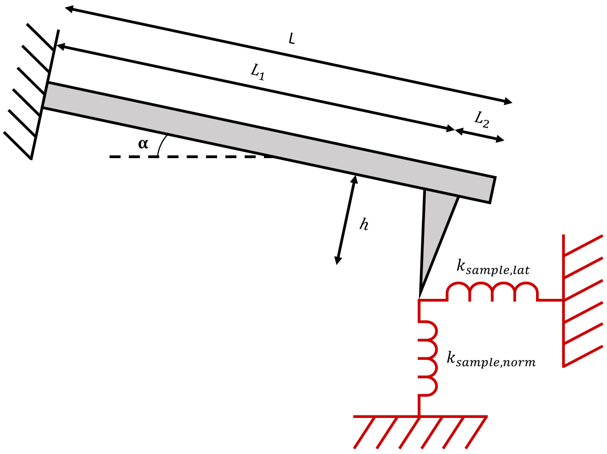

Figure S1: Modelization of clamped beam-shaped cantilever elastically coupled to a sample by its tip and used for the computation of normal sample stiffness. The cantilever characteristics, i.e. L, L_1, L_2, h and α correspond, respectively, to cantilever length, length from the clamped end to the tip, length from the tip to the free end, tip height and tilt angle. The springs $k_{sample,norm}$ and $k_{sample,lat}$ stand, respectively, for normal and lateral sample stiffnesses.

Wavenumbers x_n and y_n associated with the measured n^{th} flexural contact resonance f_n and torsional contact resonances t_n are computed by

$$x_n L = x_n^0 L \sqrt{\frac{f_n}{f_n^0}},$$

where f_n^0 is the cantilever resonance of nth flexural mode in free space and x_n^0 the associated wavelength (values given in Rabe [3]) obtained from the resolution of the characteristic equation $cos(x_nL)cosh(x_nL)+1=0$ and

$$y_n = \frac{(2n-1)\pi}{2}\frac{t_n}{t_n^0},$$

where t_n^0 is the cantilever resonance of nth torsional mode in free space.

The normalized lateral contact stiffness is determined by

$$k_{sample,lat} = -\frac{y_nLcos(y_nL)}{sin(y_nL_1)cos(y_nL_2)}k_{lat},$$

where k_{lat} is the lateral cantilever stiffness constant.

The normal contact stiffness normalized with the cantilever flexural stiffness constant k_1 is obtained from the expression

$$\frac{k_{sample,norm}}{k_1} = \frac{-B\pm\sqrt{B^2-4AC}}{6A}, \tag{1}$$

where the positive root corresponds to the normalized contact stiffness and parameters A, B and C are defined by

$$A = \frac{k_{sample,lat}}{k_{sample,norm}}A', \tag{2}$$

where

$$A' = \left(\frac{h}{L_1}\right)^2\left(1-cos(x_nL_1)cosh(x_nL_1)\right)\left(1+cos(x_nL_2)cosh(x_nL_2)\right),$$

$$B = B_1 + B_2 + B_3, \tag{3}$$

with

$$B_1 = \left[sin^2(\alpha) + \left(\frac{k_{sample,lat}}{k_{sample,norm}} \right) cos^2(\alpha) \right] B_1^{'}, \tag{4}$$

where

$$B_1^{'} = \left(\frac{h}{L_1}\right)^2 (x_n L_1)^3 \Bigg[\Big(1 + cos(x_n L_2) cosh(x_n L_2)\Big) \Big(sin(x_n L_1) cosh(x_n L_1) + cos(x_n L_1) sinh(x_n L_1) \Big)$$
$$- \Big(1 - cos(x_n L_1) cosh(x_n L_1)\Big) \Big(sin(x_n L_2) cosh(x_n L_2) + cos(x_n L_2) sinh(x_n L_2) \Big) \Bigg],$$

$$B_2 = \left[\left(\frac{k_{sample,lat}}{k_{sample,norm}} - 1 \right) cos(\alpha) sin(\alpha) \right] B_2^{'}, \tag{5}$$

where

$$B_2^{'} = 2\left(\frac{h}{L_1}\right) (x_n L_1)^2 \Bigg[\Big(1 + cos(x_n L_2) cosh(x_n L_2)\Big) sin(x_n L_1) sinh(x_n L_1)$$
$$+ \Big(1 - cos(x_n L_1) cosh(x_n L_1)\Big) sin(x_n L_2) sinh(x_n L_2) \Bigg],$$

Factor 2, that does not appear in Hurley and Turner [2], is actually a necessary correction, as introduced by Steiner et al. [5],

$$B_3 = \left[cos^2(\alpha) + \left(\frac{k_{sample,lat}}{k_{sample,norm}}\right) sin^2(\alpha)\right] B_3^{'}, \tag{6}$$

where

$$B_3^{'} = x_n L_1 \Big[\Big(1 + cos(x_n L_2) cosh(x_n L_2)\Big) \Big(sin(x_n L_1) cosh(x_n L_1) - cos(x_n L_1) sinh(x_n L_1)\Big) - \Big(1 - cos(x_n L_1) cosh(x_n L_1)\Big) \Big(sin(x_n L_2) cosh(x_n L_2) - cos(x_n L_2) sinh(x_n L_2)\Big) \Big],$$

and

$$C = 2(x_n L_1)^4 (1 + cos(x_n L) cosh(x_n L)). \tag{7}$$

Parameter B can be split into two terms, one with $k_{sample,norm}$ and the other without;

$$B = \beta_1 + \beta_2 \frac{k_{sample,lat}}{k_{sample,norm}},$$

with

$$\beta_1 = sin^2(\alpha) B_1^{'} - cos(\alpha) sin(\alpha) B_2^{'} + cos^2(\alpha) B_3^{'},$$

and

$$\beta_2 = cos^2(\alpha) B_1^{'} + cos(\alpha) sin(\alpha) B_2^{'} + sin^2(\alpha) B_3^{'}.$$

By setting a new variable ε of expression

$$\varepsilon = 6A' \frac{k_{sample,lat}}{k_1},$$

we can rewrite equation (1) as

$$\varepsilon + B = \pm\sqrt{B^2 - 4AC}.$$

Finally, by squaring each side of the equation and isolating $k_{sample,norm}$, we find the following expression for the normal sample stiffness

$$k_{sample,norm} = -\frac{2A'C\varepsilon^{-1} + \beta_2}{0.5\varepsilon + \beta_1} k_{sample,lat}.$$

References

1. Bubendorf, A. Elasticity of Polymers Investigated by Atomic Force Microscopy. Ph. D. Thesis, University of Basel, Basel, Switzerland, 2015.

2. Hurley, D. C.; Turner, J. A. *J. Appl. Phys.* **2007**, *102*, 033509. doi:10.1063/1.2767387.

3. Rabe, U. Atomic Force Acoustic Microscopy. In *Applied Scanning Probe Methods II*; Bhushan, B., Fuchs, H., Eds.; Springer: Berlin, Heidelberg, Germany, 2006; pp 37–90. doi: 10.1007/3-540-27453-7_2.

4. Rabe, U.; Turner, J. A.; Arnold, W. *Appl. Phys. A* **1998**, *66*, 277–282. doi:10.1007/s003390051145.

5. Steiner, P.; Roth, R.; Gnecco, E.; Glatzel, T.; Baratoff, A.; Meyer, E. *Nanotechnology* **2009**, *20*, 495701. doi:10.1088/0957-4484/20/49/495701.
